# Supplementary material for: Novel approach for predicting fake news stance detection using large word embedding blending and customized CNN model
Source: PLoS One. 2024 Dec 13;19(12):e0314174. doi: 10.1371/journal.pone.0314174 (PMC11643300; doi:10.1371/journal.pone.0314174)
Supplement: Attachment — Submitted filename: Response_Letter_Plos_One_abdulaziz_altamimi.pdf [file pone.0314174.s002.pdf]

Submission ID: PONE-D-24-26648

Article Title: Novel Approach For Predicting Fake News Stance Detection Using Large Word Embedding Blending and Customized CNN Model

August 8, 2024

Dear Editors,

First of all, we would like to extend our sincere gratitude to all Editors for allowing a re-submission of our manuscript, with an opportunity to address the reviewer's comments.

We have done our best to modify the paper to reflect all the comments made by the anonymous reviewers. These changes are fully addressed in this reply letter. For clarity, we have marked our responses in blue.

Please find the enclosed revised version of the paper along with the summary of the reviewer's comments. For clarity, our responses have been written in blue color. whenever we copy text from the manuscript, it is given in red color.

REVIEWER 1

Reviewer # 1 (Concern # 1)

The novel method proposed by the author combines three popular word embedding techniques. Can you describe in detail how these three techniques are combined, and present this combined way in a diagram.

First of all thank you so much for accepting this paper and pointing out this minor lack in paper. Respected reviewer, we have added steps details, pseudocode, and illustrative diagram of these word embedding combine. The added details are:

The illustration of combining features are shown in Figure 1. In the diagram, Red circles (o) represent the FastText embeddings, Green squares (s) represent the FastText Subword embeddings, Blue triangles ($\wedge$) represent the GloVe embeddings, and Black crosses (x) represent the combined embeddings. The steps that are involved in combining features are:

1. **Load pre-trained embeddings**: Ensure all embeddings are loaded correctly.
2. **Normalize embeddings**: Make sure all embeddings have the same dimensions.
3. **Combine embeddings**: Compute the weighted average of the embeddings for each word.
4. **Handle missing words**: Provide a default embedding for words not present in all embeddings.
5. **Save the combined embeddings**: Store the final embeddings for future use.

```
combined_embeddings = {}
alpha, beta, gamma = 0.33, 0.33, 0.34
```

```
for word in fasttext_embeddings:
    if word in fasttext_subword_embeddings and word in glove_embeddings:
        combined_embedding = (alpha * fasttext_embeddings[word] +
                              beta * fasttext_subword_embeddings[word] +
                              gamma * glove_embeddings[word])
        combined_embeddings[word] = combined_embedding
    # Handle words not present in all embeddings
    elif word in fasttext_subword_embeddings:
        combined_embedding = (alpha * fasttext_embeddings[word] +
                              beta * fasttext_subword_embeddings[word] +
                              gamma * np.zeros(embedding_dim))
        combined_embeddings[word] = combined_embedding
    elif word in glove_embeddings:
        combined_embedding = (alpha * fasttext_embeddings[word] +
                              beta * np.zeros(embedding_dim) +
                              gamma * glove_embeddings[word])
        combined_embeddings[word] = combined_embedding
    else:
        combined_embedding = alpha * fasttext_embeddings[word] + beta
        * np.zeros(embedding_dim) + gamma * np.zeros(embedding_dim)
        combined_embeddings[word] = combined_embedding

# Repeat similar steps for words in fasttext_subword_embeddings
and glove_embeddings that were not in fasttext_embeddings
```

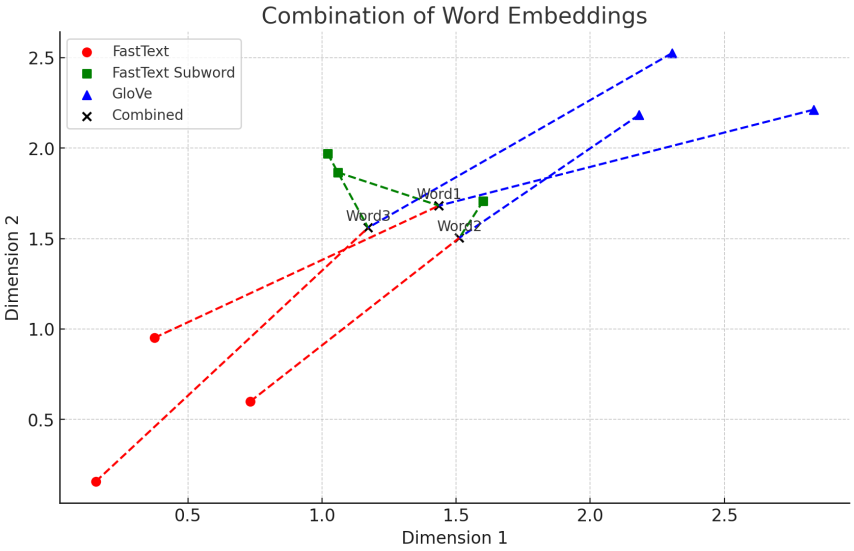

Figure 1: Illustration of word embedding fusion.

Reviewer # 1 (Concern # 2)

The authors should pay attention to the standardization of symbols, such as: 1) in subsection "3.3.3 fastText", "for" and "give", should be "for" and "give"; 2) in subsection "3.3.8 Modeling Methods", variables y, i should be italic.

Respected reviewer, we have removed these mistakes and updated the paper as suggested.

Reviewer # 1 (Concern # 3)

In subsection 4.1, comparing the experimental results of different methods, it is recommended to present them in figures for a more intuitive way.

Respected reviewer, we have added graphs of combined features complete result and accuracy comparison of all learning model with all word embedding techniques in the paper.

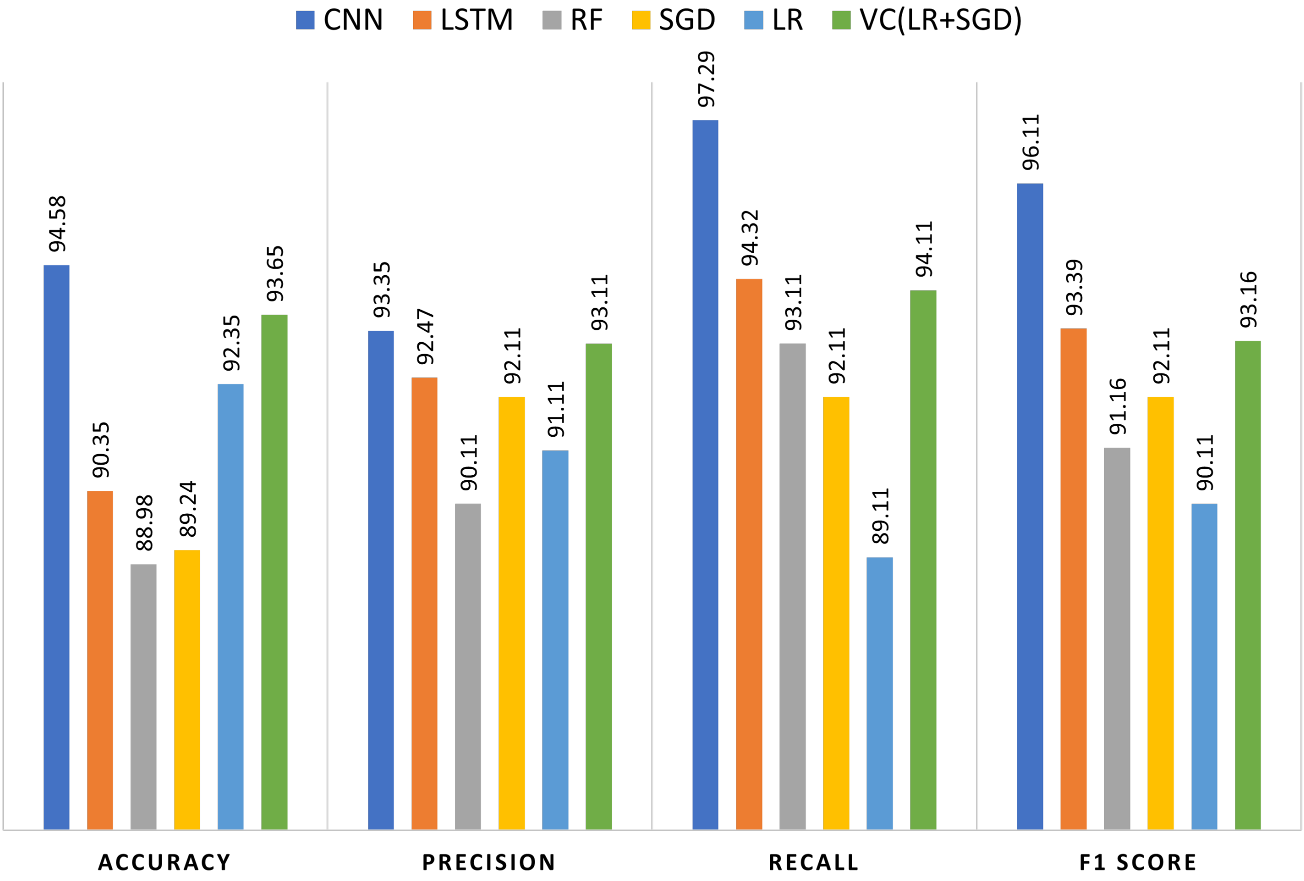

Figure 2: Comparison of all learning models using combined features.

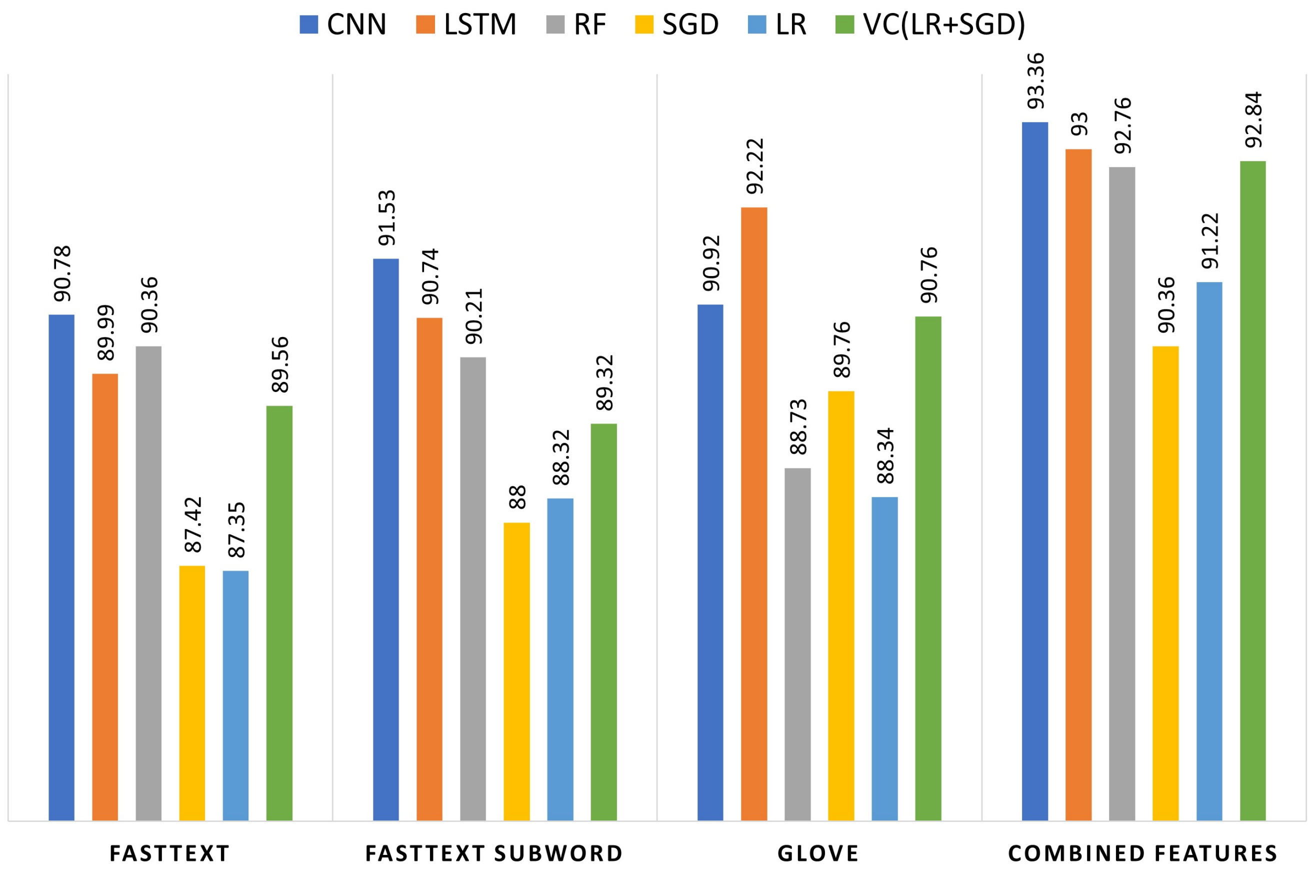

Figure 3: Comparison graph of all word embedding with all learning models.
