# Supplementary material for: Novel approach for predicting fake news stance detection using large word embedding blending and customized CNN model
Source: PLoS One. 2024 Dec 13;19(12):e0314174. doi: 10.1371/journal.pone.0314174 (PMC11643300; doi:10.1371/journal.pone.0314174)
Supplement: Attachment — Submitted filename: Response_Letter_Plos_One_abdulaziz_altamimi.pdf [file pone.0314174.s003.pdf]

Submission ID: PONE-D-24-26648R1

Article Title: Novel Approach For Predicting Fake News Stance Detection Using Large Word Embedding Blending and Customized CNN Model

October 7, 2024

Dear Editors,

First of all, we would like to extend our sincere gratitude to all Editors for allowing a re-submission of our manuscript, with an opportunity to address the reviewer's comments.

We have done our best to modify the paper to reflect all the comments made by the anonymous reviewers. These changes are fully addressed in this reply letter. For clarity, we have marked our responses in blue.

Please find the enclosed revised version of the paper along with the summary of the reviewer's comments. For clarity, our responses have been written in blue color. whenever we copy text from the manuscript, it is given in red color.

REVIEWER 2

Reviewer # 2 (Concern # 1)

The use of standard word embeddings like FastText, FastText-Subword, and GloVe, combined with CNN, is well-explored in the field of NLP. This research does not present any significant innovation in terms of methodology or model architecture.

Respected reviewer, While it is true that the combination of FastText, FastText-Subword, and GloVe embeddings with CNN has been explored in natural language processing (NLP), the novelty of this research lies in how these embeddings are blended and optimized for the specific task of fake news stance detection. Unlike traditional approaches that use a single embedding, we propose an innovative hybrid embedding strategy that maximizes the strengths of each embedding (FastText for efficient representation of words, FastText-Subword for handling out-of-vocabulary words, and GloVe for capturing global context). Furthermore, the inclusion of PCA for dimensionality reduction and feature selection enhances the model's performance by focusing on the most relevant features, reducing computational complexity, and mitigating noise. This tailored design, combined with a customized CNN architecture, enables superior performance when benchmarked against several machine learning and deep learning models, as well as independent Arabic fake news datasets, which demonstrates the framework's generalization and robustness.

Reviewer # 2 (Concern # 2)

The explanation of how the CNN model is customized is vague. The current version lacks a clear description of the specific innovations in the CNN architecture, making it difficult to assess its contribution.

Respected reviewer, The proposed CNN architecture was specifically tailored to address the complexities of fake news stance detection, especially when utilizing large, blended word embeddings such as FastText, FastText-Subword, and GloVe. This customization introduces several key innovations. First, the input layer

is designed to handle the high-dimensional combined word embeddings, allowing the model to effectively process word-level semantics and subword information simultaneously. The convolutional layer employs a larger-than-usual kernel size of 7x7, chosen to capture broader word patterns and n-gram structures across sentences, which is critical for understanding long-range word dependencies in text data. Additionally, 64 filters were selected to strike a balance between computational efficiency and the ability to extract meaningful features from the multi-embedding space.
To further enhance the architecture, 2x2 max pooling is used after the convolutional layer to reduce dimensionality, while a global max-pooling layer is introduced to select the most prominent features from each filter. This helps retain the most important information from texts of varying lengths, crucial for stance detection. To prevent overfitting and improve generalization on noisy real-world data, a dropout layer with a 0.5 rate was incorporated, randomly disabling 50% of neurons during training. Following this, a dense layer with 32 neurons acts as a decision-making layer, integrating the learned features before passing them to the output layer.
The final layer uses softmax activation to perform multi-class classification, distinguishing between three classes (agree, disagree, and discuss) within the Fake News Challenge dataset. The model is trained using a categorical cross-entropy loss function, which ensures optimal learning for the multi-class task. Key innovations, such as the larger 7x7 kernel size and global max pooling, enhance the model's ability to capture broader context and retain relevant features. Combined with dropout regularization and a well-structured dense layer, these enhancements allow the CNN to efficiently handle the intricacies of fake news stance detection, improving its performance compared to standard models. This customization offers significant improvements, particularly in handling multi-dimensional word embeddings and noisy real-world data.
If you still need further clarification, I am ready to share.

Reviewer # 2 (Concern # 3)
The model's accuracy and other performance metrics are reported, there is no discussion on the practical impact of the model, such as how well it performs under real-world conditions where data can be noisier and more complex.

Respected reviewer, While the reported accuracy and performance metrics reflect the robustness of the proposed model on benchmark datasets, we acknowledge the importance of evaluating its performance in real-world scenarios where data can be significantly noisier and more complex. In real-world conditions, news articles often contain ambiguous, misleading, or incomplete information, which could affect the model's predictions. To simulate such conditions, additional experiments are needed that I am planning to do in the future work. Future work will focus on deploying the model in real-world environments and conducting a detailed sensitivity analysis to quantify its robustness to noise, data imbalance, and evolving patterns of misinformation. This will provide deeper insights into how the model can be adapted for deployment in practical fake news detection systems.
At the revision, I have done experiments on multi-lingual dataset to show the significance of the proposed model with different types of languages.

Reviewer # 2 (Concern # 4)
The Fake News Challenge dataset is commonly used but may not fully represent the complexity of fake news detection. No details are provided on the characteristics of the Arabic Fake News dataset, raising questions about the robustness of cross-lingual performance claims.

Respected reviewer, I have evaluated my proposed model on the new dataset and added a separate subsection in the paper. The added subsection details are:

0.1 Significance of the proposed framework on on multi-lingual dataset

The TALLIP multi-lingual fake news detection dataset provides a unique opportunity to evaluate the effectiveness of fake news stance detection models across multiple languages, including English, Hindi, Indonesian, Swahili, Vietnamese, and a multilingual set. The challenge of fake news detection across different languages lies in the variations in linguistic structure, contextual understanding, and cultural

nuances. To tackle this issue, we proposed a novel approach leveraging a large word embedding blending technique integrated with a customized CNN model. The proposed model was rigorously evaluated on the TALLIP dataset, and the results were compared with several state-of-the-art models.

Table 3 presents a comparison of the accuracy achieved by various models on the TALLIP dataset, including XLM-RoBERTa, mBERT, Semantic Graph-Based Topic Modelling, Bi-directional mBERT, and our proposed model. The results show that the proposed model consistently outperformed the SOTA models across all languages. Specifically, for English, the proposed model achieved an accuracy of 89.15%, which is higher than the best-performing Semantic Graph-Based Topic Modelling model (88.58%). In Hindi, the proposed model reached 90.19%, significantly outperforming Bi-directional mBERT (89.51%) and XLM-RoBERTa (86.19%). Similarly, for Indonesian and Swahili, the proposed model achieved accuracies of 90.95% and 90.76%, respectively, which is a substantial improvement compared to the baseline models, particularly outperforming XLM-RoBERTa, which struggled on Swahili with an accuracy of only 70.29%. For Vietnamese, our model also demonstrated superior performance with an accuracy of 90.34%, compared to the highest baseline of 87.52%.

The blending of FastText, FastText-Subword, and GloVe embeddings, combined with a customized CNN architecture, allowed the proposed model to capture nuanced semantic features from the text, contributing to its superior performance. The multi-embedding approach enables the model to handle variations in vocabulary and context, which is particularly crucial for low-resource languages like Swahili and Vietnamese. Additionally, by employing PCA for dimensionality reduction and selecting significant features, the proposed model efficiently managed the complexity of the dataset, preventing overfitting and ensuring generalizability across different languages and data splits.

Table 1: Results comparison of SOTA models with respect to accuracy (%) on TALLIP multi-lingual dataset.

**Study**	**English**	**Hindi**	**Indonesian**	**Swahili**	**Vietnamese**	**Multilingual**
**XLM-RoBERTa [2]**	86.85	86.19	84.73	70.29	84.99	64.42
**mBERT [2]**	84.34	82.43	79.63	76.58	77.67	83.54
**Semantic graph-based topic modelling [4]**	88.58	86.31	88.09	83.99	83.18	NA
**Bi-directional mBERT [1]**	88.44	89.51	90.57	89.06	87.52	86.09
**Proposed**	89.15	90.19	90.95	90.76	90.34	89.64

Reviewer # 2 (Concern # 5)

The use of PCA for feature management is mentioned but not justified. How PCA contributes to the performance improvement or how much dimensionality was reduced is left unexplained, making it unclear whether this step was necessary.

Respected reviewer, I have added these lines in the paper to address your this concern.

Following PCA reduction, the dataset contains features that were present in the original data, and the covariance matrix is used to determine the principal component. Using a pool of 3400 features, PCA is used in this study to select significantly important 1500 features for the experiments.

Reviewer # 2 (Concern # 6)

The current version suggests the framework shows "superior performance" when compared with other approaches, but no substantial evidence is presented to support these claims beyond metrics. There's no detailed comparison with alternative models or baselines.

Respected reviewer, in this table I have compared my proposed model with SOTA research works.

Reviewer # 2 (Concern # 7)

While the model reportedly performs well on an Arabic dataset, there is no detailed validation process to

Table 2: Comparison with previously published studies listed in research work [3].

Model	Accuracy Performance (%)
TalosComb	82
TalosTree	83
TalosCNN	50.2
Athene	82
UCLMR	81.7
FearMLP	82.5
StackLSTM	82.1
Upperbond	85.9
Proposed model	94.58

show how it generalizes across different languages and news genres, making the cross-lingual claim questionable.

Respected reviewer, I have evaluated my proposed model on the new dataset and added a separate subsection in the paper. The added subsection details are:

0.2 Significance of the proposed framework on on multi-lingual dataset

The TALLIP multi-lingual fake news detection dataset provides a unique opportunity to evaluate the effectiveness of fake news stance detection models across multiple languages, including English, Hindi, Indonesian, Swahili, Vietnamese, and a multilingual set. The challenge of fake news detection across different languages lies in the variations in linguistic structure, contextual understanding, and cultural nuances. To tackle this issue, we proposed a novel approach leveraging a large word embedding blending technique integrated with a customized CNN model. The proposed model was rigorously evaluated on the TALLIP dataset, and the results were compared with several state-of-the-art models.

Table 3 presents a comparison of the accuracy achieved by various models on the TALLIP dataset, including XLM-RoBERTa, mBERT, Semantic Graph-Based Topic Modelling, Bi-directional mBERT, and our proposed model. The results show that the proposed model consistently outperformed the SOTA models across all languages. Specifically, for English, the proposed model achieved an accuracy of 89.15%, which is higher than the best-performing Semantic Graph-Based Topic Modelling model (88.58%). In Hindi, the proposed model reached 90.19%, significantly outperforming Bi-directional mBERT (89.51%) and XLM-RoBERTa (86.19%). Similarly, for Indonesian and Swahili, the proposed model achieved accuracies of 90.95% and 90.76%, respectively, which is a substantial improvement compared to the baseline models, particularly outperforming XLM-RoBERTa, which struggled on Swahili with an accuracy of only 70.29%. For Vietnamese, our model also demonstrated superior performance with an accuracy of 90.34%, compared to the highest baseline of 87.52%.

The blending of FastText, FastText-Subword, and GloVe embeddings, combined with a customized CNN architecture, allowed the proposed model to capture nuanced semantic features from the text, contributing to its superior performance. The multi-embedding approach enables the model to handle variations in vocabulary and context, which is particularly crucial for low-resource languages like Swahili and Vietnamese. Additionally, by employing PCA for dimensionality reduction and selecting significant features, the proposed model efficiently managed the complexity of the dataset, preventing overfitting and ensuring generalizability across different languages and data splits.

REVIEWER 3

Reviewer # 3 (Concern # 1)

The language of the paper should be improved. Tools like Grammarly can be helpful. Some example statements that have punctuation and grammar mistakes:

Table 3: Results comparison of SOTA models with respect to accuracy (%) on TALLIP multi-lingual dataset.

Study	English	Hindi	Indonesian	Swahili	Vietnamese	Multilingual
**XLM-RoBERTa [2]**	86.85	86.19	84.73	70.29	84.99	64.42
**mBERT [2]**	84.34	82.43	79.63	76.58	77.67	83.54
**Semantic graph-based topic modelling [4]**	88.58	86.31	88.09	83.99	83.18	NA
**Bi-directional mBERT [1]**	88.44	89.51	90.57	89.06	87.52	86.09
**Proposed**	89.15	90.19	90.95	90.76	90.34	89.64

a. The pernicious impact of such misinformation [2]. hits all sectors of society.
b. This study uses ML models for the accurate classification of fake news stances on social media. the main contribution of the study is
c. This stage in the process of processing natural language is crucial.

Respected reviewer, I have proofread the complete paper on my own as well as using your suggested tools. Now the paper quality has improved a lot by removing typos, and grammatical, and sentence structure mistakes.

Reviewer # 3 (Concern # 2)

Table 4 metrics are not clear. The column titles can be modified as below: Accuracy (%) Precision (%) Recall (%) F1-Score (%)

Respected reviewer, I have updated the tables as per your suggestions to improve the readability of the paper.

Reviewer # 3 (Concern # 3)

For Table 5, 6,7, 8, and 9 the % characters can be moved to the column titles. That would improve the readability of the tables. For example, the column titles of Table 9 can be as below: Models fastText (%) fastText-Subword (%) GloVe (%) Combined-Features (%) Then, the % signs next to the result numbers can be removed.

Respected reviewer, I have updated the tables as per your suggestions to improve the readability of the paper.

Reviewer # 3 (Concern # 4)

To improve the contribution to future research, the used code can be uploaded to a platform like GitHub, and a link can be provided in the paper.

Respected reviewer, I have added my proposed framework code in the submission portal of Plos One. In my declaration, I have clearly stats that I will share complete working code along with dataset when the paper is accepted for publication.

References

[1] J. Alghamdi, Y. Lin, and S. Luo. Fake news detection in low-resource languages: A novel hybrid summarization approach. *Knowledge-Based Systems*, 296:111884, 2024.

[2] A. De, D. Bandyopadhyay, B. Gain, and A. Ekbal. A transformer-based approach to multilingual fake news detection in low-resource languages. *Transactions on Asian and Low-Resource Language Information Processing*, 21(1):1–20, 2021.

[3] A. Hanselowski, A. PVS, B. Schiller, F. Caspelherr, D. Chaudhuri, C. M. Meyer, and I. Gurevych. A retrospective analysis of the fake news challenge stance detection task. *arXiv preprint arXiv:1806.05180*, 2018.

[4] R. Mohawesh, X. Liu, H. M. Arini, Y. Wu, and H. Yin. Semantic graph based topic modelling framework for multilingual fake news detection. *AI Open*, 4:33–41, 2023.
